# Optimizing the ordering of the Hadamard masks of ghost imaging suitable for the efficient face reconstruction using the max-projection method

**DOI:** 10.1038/s41598-023-48453-2

**Published:** 2023-12-19

**Authors:** Haipeng Zhang, Kang Du, Changzhe Zhao, Jie Tang, Shangyu Si, Wenhong Jia, Lian Xue, Zhongliang Li

**Affiliations:** 1 Shanghai Synchrotron Radiation Facility, Shanghai Advanced Research Institute, Chinese Academy of Sciences, Shanghai, 201204 China; 2 Shanghai Institute of Applied Physics, Chinese Academy of Sciences, Shanghai, 201800 China; 3 University of Chinese Academy of Sciences, Beijing, 100049 China

**Keywords:** Imaging and sensing, Quantum optics

## Abstract

One crucial component of ghost imaging (GI) is the encoded mask. Higher-quality reconstruction at lower sampling rates is still a major challenge for GI. Inspired by deep learning, max-projection method is proposed in the paper to reorder the Hadamard masks for its efficient and rapid reconstruction. The simulations demonstrated that max-projection ordering with only 20 face training images yielded excellent reconstruction outcomes. In noise-free simulations, at an ultralow sampling rate of 5%, the PSNR of the max-projection ordering was 1.1 dB higher than that of the cake-cutting ordering with the best performance in the reference group. In noisy simulations, at ultralow sampling rates, the retrieved images remained almost identical to their noise-free counterparts. Irrespective of the presence or absence of noise, the max-projection ordering guaranteed the highest fidelity of image reconstruction at ultralow sampling rates. The reconstruction time was reduced to mere milliseconds, thereby enabling swift visualization of dynamic phenomena. Accordingly, the max-projection ordering Hadamard matrix offers a promising solution for real-time GI due to its higher reconstruction quality, stronger noise immunity and millisecond reconstruction time.

## Introduction

Ghost imaging (GI)^1–6^, also referred to as single-pixel imaging (SPI)^7–14^, represents a novel nonlocalized imaging method that distinguishes itself from conventional direct imaging. GI can overcome the challenge of traditional multipixel imaging that it is technologically unavailable under harsh circumstances, including poor illumination^15^ and turbulent environments^16^, and it can obtain a spatial resolution beyond the Rayleigh diffraction limit^17^. One pivotal component of the commonly employed GI system is a series of encoded masks meticulously arranged in a specific sequence to modulate the light fields incident upon the object (also named structural illumination^7^). In principle, the fundamental factors that influence the efficacy of GI can be attributed to the selection of modulation masks^8–10,18,19^ and reconstruction methods^20–22^.

To achieve high-fidelity reconstruction at sub-Nyquist sampling, the modulated masks are generally encoded as random matrices, Hadamard matrices^8,18,19^, Fourier matrices^9^ and wavelet matrices^10^ to satisfy the restricted isometry property (RIP)^23,24^ of compressed sensing (CS) theory. Although the reconstruction quality is also constrained by the noise level and the sparsity of the object, higher sampling rates still bring a better signal-to-noise ratio (SNR). However, the pursuit of higher sampling rates will result in longer data acquisition and computation time, which hinders the development of the real-time GI. Hence, the challenge of quickly retrieving high-SNR images at ultralow sampling rates needs to be addressed immediately.

Taking programmability, modulation speed and price into consideration, Hadamard masks stand out because the frame rate of the Hadamard masks generated by digital micromirror devices (DMDs) can reach tens of kilohertz^14,25^, which is beneficial to the development of real-time GI. Additionally, the orthogonal and binary superiority of the Hadamard masks makes it highly robust to background noise, consequently reducing the requisite sampling rate. Moreover, image reconstruction can be further improved through a differential measurement of the Hadamard matrix, which diminishes the negative effect of the environmental noise^26^. As a result, the Hadamard matrix has gained unparalleled popularity as the predominant choice for modulation masks in GI applications.

It is imperative to emphasize that based on different orderings of the Hadamard basis, there are significant differences in the fidelity of ‘ghost’ images recovered at subsampling^12^. Therefore, researchers have successively proposed diverse ordering schemes for the Hadamard masks to enhance the reconstruction quality at ultralow sampling rates. By using the Walsh-Hadamard transform on the Walsh-ordering Hadamard matrix, the algorithm not only exhibits exceptional efficiency but is also easy to manipulate in hardware. Moreover, when implementing ‘Russian Doll’ (RD) ordering, Hadamard patterns^19^ can reproduce an image resembling the object with merely 6% of the measurements required by the Nyquist sampling theorem. In comparison to RD ordering, ‘cake-cutting’ (CC)^18^ and ‘origami pattern’^8^ sorting approaches can achieve higher reconstruction fidelity of natural images with fewer measurements. Drawing on the comprehension of natural images’ characteristics, a novel ordering of Hadamard masks sorted by the total variation ascending order is put forward and yields remarkable advancement in sub-Nyquist sampling SPI^27^.

With the rapid development of deep neural networks^13^, their significant superiority in uncovering the features within structured data or images has garnered tremendous success across various scientific and technological domains. Deep learning-based automatic sorting of the Hadamard basis is considered a promising way to efficiently solve the underdetermination problem with prior knowledge, thereby enabling real-time GI with enhanced quality. Inspired by the idea of deep learning to excavate and utilize image features, max-projection ordering is put forward in this paper to seek better-ordering Hadamard masks suitable for natural images possessing uniform features, such as face images or in the thriving area of cerebral imaging where many similar neural images exist^28–30^. Furthermore, the orthogonality of the Hadamard matrix can be used to simplify the computation of its Moore–Penrose inverse, which speeds up image reconstruction. The max-projection ordering of the Hadamard masks is expected to yield a higher-fidelity reconstruction with fewer measurements, thus becoming a promising solution for the advancement of real-time GI.

## Theory

### The max-projection method to optimize the ordering of the Hadamard masks

Unlike conventional GI, differential GI^20^ and normalized GI^31^ which rely on ensemble averaging of the intensity, David et al*.*^32^ orthogonalized the random-matrix basis via the Gram‒Schmidt process and calculated the corresponding weighting coefficients for each individual orthogonal basis, so that the object can be obtained efficiently through the linear representation of these orthogonal bases:

1 $$f=\sum_{k=1}^{N}{\widetilde{w}}_{k}{\widetilde{R}}_{k}\approx \sum_{k=1}^{M}{\widetilde{w}}_{k}{\widetilde{R}}_{k}\equiv {f}^{(M)}$$

where ${\widetilde{w}}_{k}=\langle f,{\widetilde{R}}_{k}\rangle$ is the weight coefficient of the corresponding orthogonal basis ${\widetilde{R}}_{k}$. Furthermore, ${f}^{(M)}$ is the ${M}^{th}$ approximation of the ground truth $f$ projected onto the incomplete M-element subset which is the part of the complete orthogonal set $\left\{{\widetilde{R}}_{k}\right\}$ comprising N elements. Therefore, Eq. (1) can be aptly referred to as the equation of the linear combination. Inspired by this idea, a finite set of strictly orthogonal bases are selected as the masks of GI rather than the random matrix which needs to be preprocessed. The binary and orthogonal superiority inherent in Hadamard matrix renders themselves amenable to easy manufacturing and strongly resistant to noise; hence, Hadamard matrix have become popular in the mask fabrication suitable for GI. Derived from Formula (1), the objective is to seek the incomplete M-member Hadamard subset that maximizes the projection of the target image $f$. The optimal ordering for a specific image $f$ with known structural information is called the ideal ordering, where the discrepancy ${\Vert f-{f}^{(M)}\Vert }^{2}$ is minimized for the M-member Hadamard subset.

In principle, the projection of the target image on the Hadamard basis can be utilized to sort the Hadamard basis. However, the structural information of the target image is almost unknown. Inspired by the idea of deep learning, the features of the target image can be revealed through training images with similar features. Acquiring a predetermined number of orthogonal Hadamard bases onto which the training set exhibits maximum projection poses a formidable challenge. Nonetheless, it is relatively straightforward to identify a single Hadamard basis that contributes the most to all the training images. In other words, the Hadamard basis on which the entire training set $T$ has the maximum projection can be easily selected. The max-projection equation can be expressed mathematically as follows:

2 $${H}_{{k}_{1}}=\underset{{H}_{k}}{{\text{max}}}{\Vert T*{H}_{k}\Vert }_{2}^{2}$$

where $T\in {\mathbb{R}}^{S\times N}$ represents the training set aimed at optimizing the ordering of the Hadamard basis. ${H}_{k}\in {\mathbb{R}}^{N\times 1}$ is the ${k}_{th}$ row of the Hadamard matrix in its natural ordering. Each row of $T$ stands for a normalized image with similar features to the target image with $N=n\times n$ pixels. Through the max-projection approach, the Hadamard basis that contributes the most is marked as ${H}_{{k}_{1}}$. The residual term ${T}_{1}$ of $T$ after the projection on ${H}_{{k}_{1}}$ is calculated as follows:

3 $${T}_{1}^{\mathrm{^{\prime}}}={T}^{\mathrm{^{\prime}}}-{\varvec{d}}{\varvec{i}}{\varvec{a}}{\varvec{g}}\left(T{*H}_{{k}_{1}}\right)*{H}_{{k}_{1}}/n$$

where the superscript ${}{\prime}$ denotes the matrix transpose and the operator ${\varvec{d}}{\varvec{i}}{\varvec{a}}{\varvec{g}}$ signifies the operation that reconfigures the vector into a diagonal square matrix whose diagonal elements correspond to the vector. It is obvious that each row of the residual term ${T}_{1}$ is orthogonal to ${H}_{{k}_{1}}$ after the residual formula expressed as Eq. (3).

Numerous alternating calculations are performed between formulas (2) and (3), as follows:

4a $${H}_{{k}_{j}}=\underset{{H}_{k}}{{\text{max}}}{\Vert {T}_{j-1}{*H}_{k}\Vert }_{2}^{2}$$

4b $${T}_{j}^{\mathrm{^{\prime}}}={T}_{j-1}^{\mathrm{^{\prime}}}-{\varvec{d}}{\varvec{i}}{\varvec{a}}{\varvec{g}}\left({T}_{j-1}{*H}_{{k}_{j}}\right)*{H}_{{k}_{j}}/n$$

Then, a set of Hadamard bases can be obtained in accordance with the contribution degree. To distinguish it from the Hadamard matrix $H$ of the natural ordering, the rearranged Hadamard matrix is denoted as $\widetilde{H}$ according to the max-projection sorting method. In the subsampling GI, the measurement matrix $A$ is acquired from the first M orthogonal bases of $\widetilde{H}$, where bases of more significance are ranked first.

### A simple method for fast reconstruction through the Moore–Penrose pseudo-inverse of the Hadamard matrices

In theory, the orthogonal Hadamard matrix contains binary elements 1 and − 1, but in fact, the intensity distribution modulated by the masks must not be lower than zero on the object plane. Therefore, the real Hadamard matrix $\widehat{H}$ has binary elements 1 and 0 in practical applications, which results in the loss of the orthogonality of the Hadamard-type masks and increases the difficulty of solving the underdetermined problem in image reconstruction. An extremely simple method is developed to address the issue, illustrated as follows:

5 $$\widehat{H}*f=B$$

where $B\in {\mathbb{R}}^{M\times 1}$ indicates the bucket signals recorded by the single-pixel detector in the object arm. The first row of the Hadamard matrix in different orderings, including the natural ordering, the Walsh ordering, and the cake-cutting ordering, is generally the vector whose members are all ones, so the special row is denoted as ${H}_{1}$ in the paper. ${B}_{1}={H}_{1}*f$ is the first element in the observation vector $B$. The orthogonal transform equation mathematically equivalent to Eq. (5) is expressed as follows:

6 $$\left(2*\widehat{H}-{H}_{1}\right)*f=2*B-{B}_{1}$$

where the mathematical symbol “−” signifies the subtraction of the row vector ${H}_{1}$ from each row of $2*\widehat{H}$. It is evident that $H=2*\widehat{H}-{H}_{1}$ is the orthogonal Hadamard matrix that contains elements 1 and − 1. Based on the orthogonal property of the Hadamard matrix, the object information $f$ can be promptly and efficiently obtained according to the pseudo-inverse GI^33^, as shown in the reconstruction equation outlined below.

7 $$f={\left(2*\widehat{H}-{H}_{1}\right)}^{\prime}*(2*B-{B}_{1})/N$$

This simple method circumvents the computational complexity associated with the orthogonal process and reduces the large time consumption for image reconstruction. In addition to the Hadamard matrix of the orderings mentioned above, the Hadamard matrix sorted by the max-projection method and ideal ordering also situate ${H}_{1}$ in the first row because the grayscales of all the natural images exceed zero. Upon further consideration, the orthogonal Hadamard matrix, which contains 1 and − 1, shares the same sorted index sequence $\left\{{k}_{1},{k}_{2},\cdots {k}_{M}\right\}$ as the nonorthogonal Hadamard matrix of elements 1 and 0 in accordance with the max-projection idea.

## Numerical simulation

### Comparisons of face reconstruction using different orderings of the Hadamard basis

To demonstrate the generalization ability of the max-projection sorting method proposed in “The max-projection method to optimize the ordering of the Hadamard masks” section, numerical simulations were carried out to recover a random face image outside the training set. The reconstruction algorithm was chosen as the simple method described in “A simple method for fast reconstruction through the Moore-Penrose pseudo-inverse of the Hadamard matrices” section, which took full advantage of the orthogonality of the Hadamard bases and resulted in a substantial reduction in the computation time. The size of the target image was set as $64\times 64$ pixels. Moreover, the grayscales of the target image were normalized to the range of 0 to 1, as were the grayscales of all the images in the training set meeting the requirements of deep learning.

**Figure 1:**
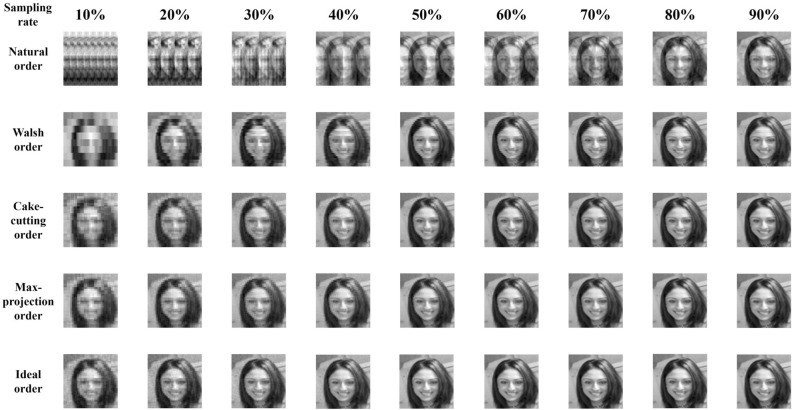
Comparisons between the face reconstructions under the Hadamard matrix for different orderings and different sampling rates.

As depicted in Fig. 1, the reconstruction quality based on the Hadamard basis of different orderings gradually increased with increasing sampling rate, which aligned with common intuition. The overlapping artifacts caused by the natural ordering severely deteriorated the reconstruction quality. Block-like artifacts were introduced into the reconstruction image based on the Walsh ordering at a low sampling rate. When the sampling rate fell below 30%, compared with the three orderings other than the ideal ordering, there was a discernible improvement in the image quality based on the max-projection ordering through explicit observation. Moreover, the images reconstructed from the max-projection ordering bore the closest resemblance to those from the ideal ordering. In particular, at a sampling rate of 10%, there were no obvious block-like artifacts on the face images recovered based on either the max-projection ordering or the ideal ordering, whereas the artifacts caused by the other three orderings resulted in the inability to distinguish the characteristic parts such as the mouth and the eyes on the faces. As the sampling rate approached 1 defined by the Nyquist sampling theorem, the reconstructed images based on all the orderings were very close to the ground truth. The reconstruction fidelity resulted from such high sampling rates made it no longer possible for the naked eye to distinguish between the reconstruction results.

**Figure 2:**
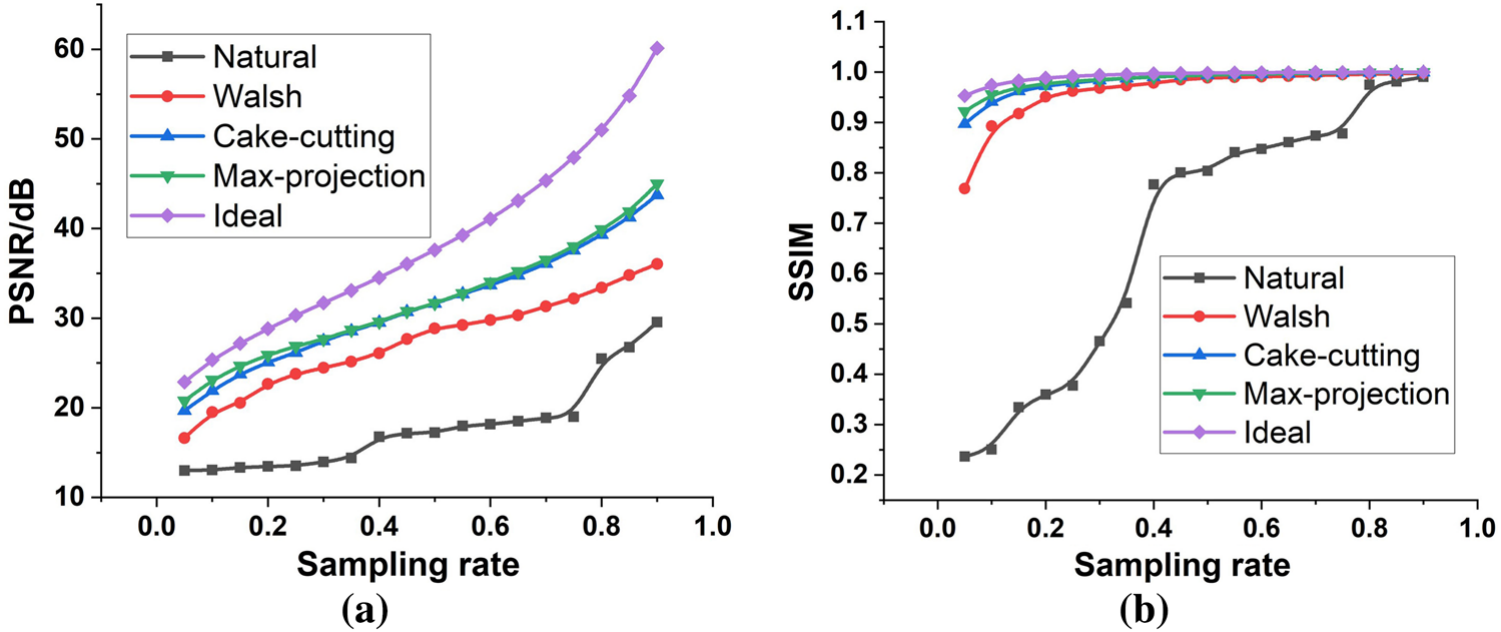
Quantitative comparisons of face reconstruction quality, including PSNR (**a**) and SSIM (**b**), under Hadamard matrices of different orderings and different sampling rates.

The quality metrics, including the structural similarity (SSIM) index and peak signal-to-noise ratio (PSNR), were utilized to evaluate the reconstruction performance based on the Hadamard basis of different orderings. As shown in Fig. 2, both the SSIM and PSNR gradually increased with increasing sampling rate in the absence of background noise, which remained consistent with the direct observation in Fig. 1. The quality of the retrieved images based on the natural ordering and Walsh ordering was clearly lower than that based on the cake-cutting ordering and the max-projection ordering. Under an ultralow sampling rate of $5\%$, the PSNRs and SSIMs of the images based on the max-projection sorting were $1.1 \; \text{dB}$ and $0.025$ higher, respectively, than those based on the cake-cutting ordering, which was the best in the reference group. At this sampling rate, the PSNRs and SSIMs of the images recovered from the ideal ordering were 2.1 dB and $0.031$ higher than those of our proposed ordering. This reflected the fact that the structural information of the target image could not be accurately characterized through the training set of face images with similar features. However, the ideal ordering could only be achieved under the precondition that the structural information of the target image was known in advance. From the preceding analysis, it can be seen that the first few bases of the Hadamard matrix sorted by the max-projection method contributed more to image reconstruction because the qualities of the reconstructed images obtained through the max-projection ordering were the closest to those obtained through the ideal ordering.

With PNSR as the metric to assess the reconstruction quality, the difference between the reconstruction quality obtained through the max-projection ordering and through the cake-cutting ordering was maximal at an ultralow sampling rate, whereas the PSNR difference was minimal between the images obtained through the ideal ordering and through the max-projection ordering; this indicates that the reconstruction quality could be greatly improved based on the Hadamard bases optimized by the max-projection method at ultralow sampling rates. The difference gradually decreased until the sampling rate increased to $40\%$ and then rapidly increased after the sampling rate reached 60%. It can be concluded that the first 40% to 60% of the Hadamard bases sorted by the cake-cutting ordering and by the max-projection ordering form an orthogonal subset possessing almost the same members, which resulted in almost the same reconstruction quality for both orderings at the sampling rates of this interval. On the other hand, the binarity of the Hadamard matrix restricted the performance of the max-projection ordering for face reconstruction, making it marginally better than that of the cake-cutting ordering generalized to all natural images. Moreover, the max-projection ordering at sampling rates of 60% to 90% showed a notable improvement compared with the cake-cutting ordering because the gap between their PSNRs obviously widened in this range of sampling rates. As the sampling rate increased from 60 to 90%, the quality divergence gradually increased between the images reconstructed by the ideal ordering and by the max-projection ordering. The discrepancy between the training set and the target image prevented the max-projection ordering from perfectly sorting the Hadamard masks according to the contribution degree.

Taking SSIM to characterize the reconstruction quality, the disparities in the reconstruction quality between the max-projection ordering and the other orderings (excluding the ideal ordering) were the most pronounced at ultralow sampling rates. As the sampling rate gradually increased to nearly 1, the reconstructions’ SSIM for the max-projection ordering approached the theoretical maximum of 1 and ranked at the top among all the orderings (excluding the ideal ordering), although the gaps between the orderings gradually shrank. The reason that the SSIM gaps shrank with the increasing sampling rate was because there was an upper limit to SSIM.

In essence, the reconstruction quality is determined by the discrepancy ${\Vert f-{f}^{(M)}\Vert }^{2}$. The smaller the discrepancy, the higher the reconstruction fidelity. Due to the training images with the similar structures to the target image, the max-projection ordering can achieve the smallest discrepancy compared with the three orderings (excluding the ideal ordering). The clarity of the reconstruction images also reflected the superiority of the max-projection ordering at low sampling rates.

In summary, both of the quality metrics indicated that the max-projection ordering yielded the optimal quality and the largest quality gap with the other orderings (excluding the ideal ordering) at the lowest sampling rate, which proved that the max-projection ordering could rank the Hadamard bases with the larger contributions at the top.

**Figure 3:**
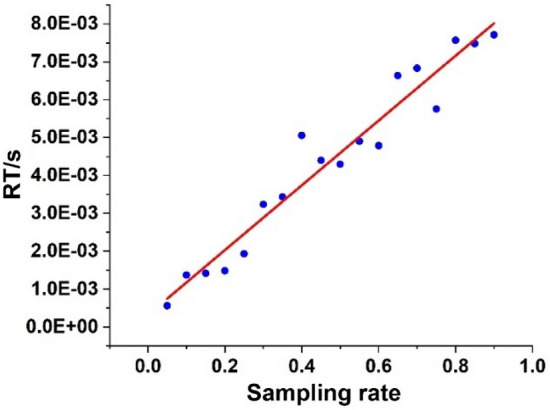
Reconstruction time (RT) at different sampling rates.

Another important metric of the algorithm performance was the time consumption for image reconstruction. As illustrated in Fig. 3, although the reconstruction time was proportional to the sampling rate, it merely took milliseconds to reconstruct an image of 64 * 64 pixels through the Moore–Penrose inverse of the Hadamard matrix because the Moore–Penrose inverse of the Hadamard matrix was its transpose, while the reconstruction based on compressed sensing or orthogonal ghost imaging^32^ generally took seconds or even more. Combined with the max-projection reordering, a high-quality reconstruction could be obtained through simple matrix multiplication rather than multiple iterations, which was of great significance for the development of real-time GI.

### Influence of the training set size on the face reconstruction quality

**Figure 4:**
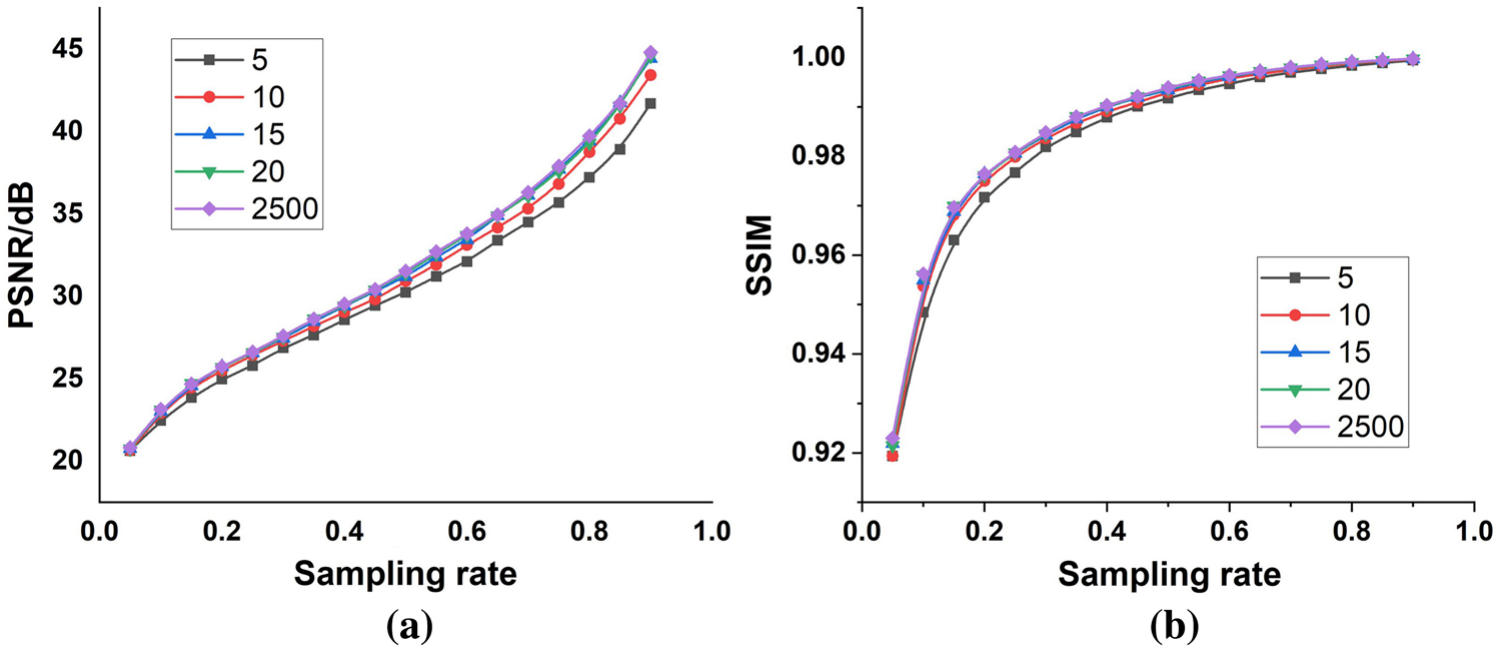
Influence of the training set size (the number of images in the training set is 5, 10, 15, 20, and 2500) on face reconstruction quality. (**a**) The PSNRs and (**b**) the SSIMs of the images reconstructed by the max-projection ordering with training sets of different sizes.

It was intuitively believed that a sizable training set was essential for reordering the Hadamard bases. However, far fewer training images than expected were needed to obtain an excellent ordering through the max-projection method. This is because the Hadamard bases had only two elements, 0 and 1, with a large gradient span. The projection maximum was limited by the binarity of the Hadamard basis, so the improved orthogonal bases with continuous grayscale variation would be selected to yield a larger projection if microfabrication gained breakthroughs in the future. As shown in Fig. 4, both the PSNR and SSIM increased with the increasing size of the training set, which was consistent with our intuition. However, at diverse sampling rates, the PSNRs and SSIMs were very close to each other at different numbers of training images, including 15, 20 and 2500, which indicated that an excellent ordering of the Hadamard bases required only a small number of training images. This conclusion is of great value in practical applications where a lack of training data is common.

### The robustness of the max-projection ordering against different levels of noise at ultralow sampling rates

In practical experiments, background noise is mainly additive Gaussian white noise introduced by the detector. Therefore, it was necessary to ascertain the noise resistance of the reconstruction algorithm based on the max-projection ordering trained with only 20 facial images. In the absence of noise interference, the speckle-distribution pattern in the reference arm was denoted as ${I}_{j}({x}_{1},{y}_{1})$*,* which represents the ${j}_{th}$ row of the max-projection ordering Hadamard matrix consisting of the elements 0 and 1; the total intensity received by the bucket detector in the signal arm was defined as ${B}_{j}={\sum }_{{x}_{2},{y}_{2}}f\left({x}_{2},{y}_{2}\right)*I({x}_{2},{y}_{2})$, where $({x}_{k},{y}_{k})(k=\mathrm{1,2})$ indicated the position coordinates on the detector plane and $f\left({x}_{2},{y}_{2}\right)$ was the intensity transmittance function of the target object to be tested. In the presence of additive noise, the speckle pattern changed to ${I}_{j}^{no}\left({x}_{1},{y}_{1}\right)={I}_{j}\left({x}_{1},{y}_{1}\right)+{N}_{1}({x}_{1},{y}_{1})$, and the corresponding bucket signal was equivalent to ${B}_{j}^{no}={\sum }_{{x}_{2},{y}_{2}}f\left({x}_{2},{y}_{2}\right)*{I}_{j}\left({x}_{2},{y}_{2}\right)+{N}_{2}({x}_{2},{y}_{2})$, where the superscript ${\prime}no{\prime}$ stood for noise and ${N}_{k}(k=\mathrm{1,2})$ indicated the same type of Gaussian noise with mean $\mu$ and standard deviation $\sigma$.

The mean $\mu$ of the additive environmental noise could generally be measured during the experimental procedures. The influence exerted by the mean $\mu$ can be eliminated through the subtraction of the mean from the measured data, so $\mu$ was usually set to 0 in the numerical simulations, so the noise level was evaluated by the standard deviation $\sigma$. The noise level of the detector was quantitatively defined as the ratio of the standard deviation $\sigma$ to the maximum dynamic range of the detector. In this simulation, due to the detector’s dynamic range of 16-bit depth, the noise level could be determined as $\sigma /65535$.

In daily experiments, the level of thermal noise introduced by the detector was usually less than ${10}^{-4}$. To demonstrate the superiority of the max-projection ordering Hadamard bases as a designed mask, the noise level of the numerical simulation was selected as ${2*10}^{-3},4*{10}^{-3},{8*10}^{-3}$ and 0, indicating various scenarios, including the absence of noise as a reference. In the presence of background noise, the underdetermined equation was established as Eq. (8) according to the intrinsic relationship between the speckle patterns and the bucket signals:

8 $${A}^{no}*f={B}^{no}$$

where the ${j}_{th}$ row of the noisy measurement matrix ${A}^{no}\in {\mathbb{R}}^{M\times N}$ is ${A}_{j}^{no}$, which is a row vector rearranged from the noisy pattern ${I}_{j}^{no}\left({x}_{1},{y}_{1}\right)$. The $jth$ element of the noisy observation vector ${B}^{no}\in {\mathbb{R}}^{M\times 1}$ is ${B}_{j}^{no}$. Referring to Eq. (7), the corresponding solution is expressed as follows:

9 $$f={(2*{A}^{no}-{A}_{1}^{no})}^{\mathrm{^{\prime}}}*(2*{B}^{no}-{B}_{1}^{no})/N$$

Due to the existence of noise, $2*{A}^{no}-{A}_{1}^{no}$ was no longer part of the perfect orthogonal Hadamard matrix, especially the 0-value region, which was severely contaminated by noise, resulting in reconstruction failure through Eq. (9). However, $2*{A}^{no}-{A}_{1}^{no}$ could be substituted with the corresponding perfect counterpart $R$ extracted from the max-ordering Hadamard matrix $\widetilde{H}$, and the solution, named the replacement reconstruction, is shown as:

10 $$f={R}^{\mathrm{^{\prime}}}*(2*{B}^{no}-{B}_{1}^{no})/N$$

**Figure 5:**
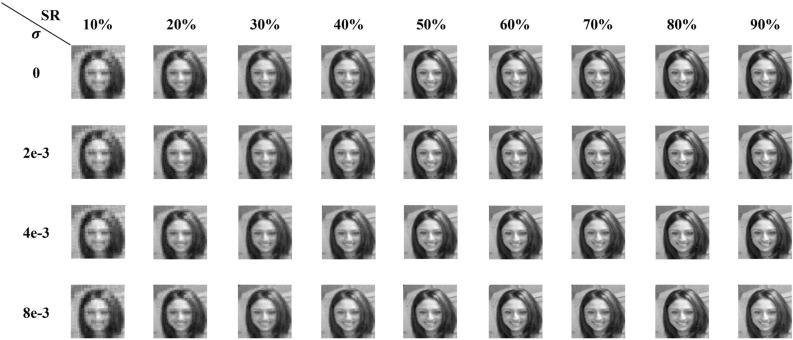
Human faces reconstructed through the replacement reconstruction method based on the max-projection ordering Hadamard bases at different sampling rates under different noise levels.

Figure 5 showed the result by the replacement reconstruction based on the max-projection ordering Hadamard bases at different sampling rates and different noise levels. It was evident that better fidelity of the reconstructed image was achieved with increasing sampling rates. Moreover, the reconstruction quality gradually deteriorated with increasing noise at a high sampling rate, while the quality changed little at an ultralow sampling rate with noise variation.

**Figure 6:**
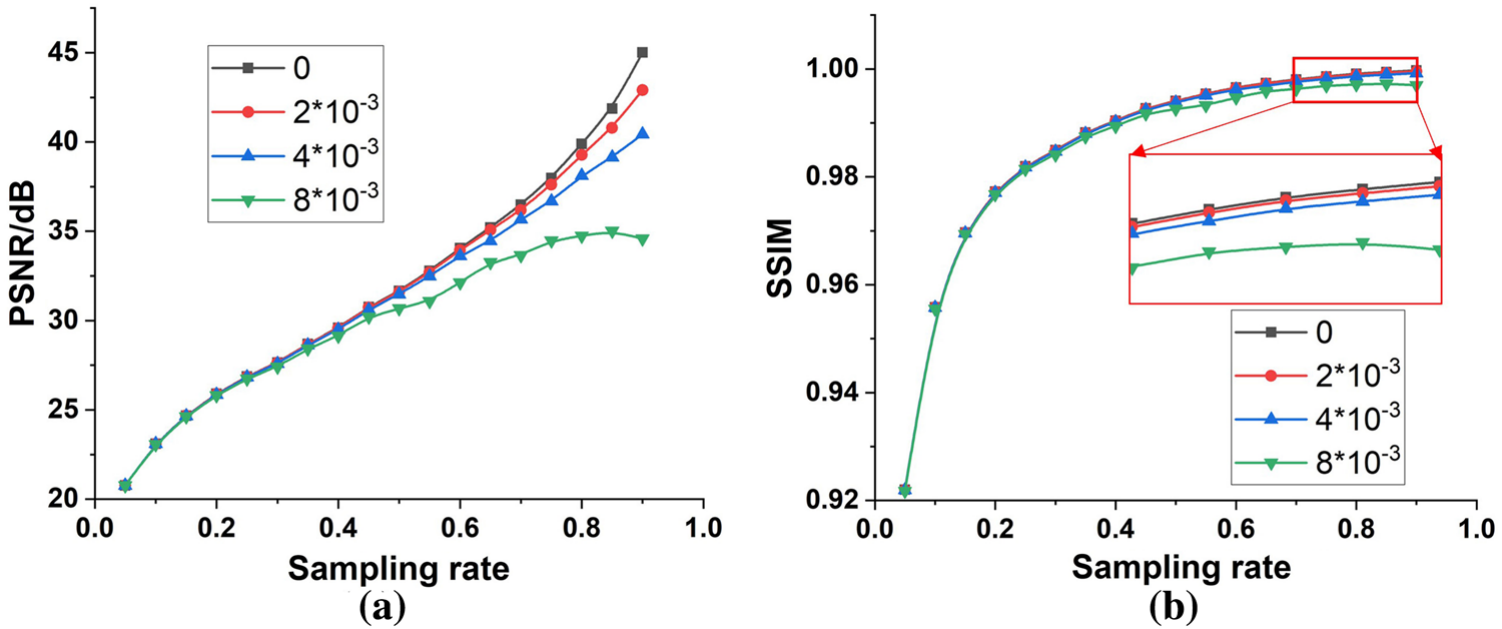
PSNR (**a**) and SSIM (**b**) of human faces reconstructed through the replacement reconstruction method based on the max-projection ordering Hadamard bases at different sampling rates and different levels of noise.

To quantitatively evaluate the reconstruction precision of human faces, the PSNR and SSIM were again employed as the quality metrics. It can be more explicitly seen from Fig. 6 that both the PSNRs and SSIMs improved as the sampling rate increased, which maintained consistency with the conclusion derived from Fig. 5. At a noise level of ${2*10}^{-3}$, much greater than the actual noise level of ${10}^{-4}$, the PSNR and SSIM curves closely resembled those without noise, which proved the strong noise resistance of the max-projection ordering Hadamard bases. In addition, the PSNR curves of different noise levels (including no noise) overlapped at low sampling rates but bifurcated more at higher sampling rates, as did the SSIM curves. Even at the highest sampling rate, the curves at a noise level of ${8*10}^{-3}$ exhibited a downward tendency. This phenomenon indicates that the more the Hadamard basis contributed to the reconstruction of the human face, the stronger its capability to resist noise; therefore, the max-projection ordering Hadamard matrix where a basis of greater significance was given more priority achieved better reconstruction fidelity at a lower sampling rate.

## Discussion and conclusions

Inspired by the concept of deep learning, the max-projection method is proposed in this paper to reorder the Hadamard bases for fast and efficient reconstruction. Numerical simulation was carried out to demonstrate the superiority of the max-projection method. In noise-free simulations, the max-projection ordering showed the greatest improvement in reconstruction precision at an ultralow sampling rate compared to the other three orderings, including the natural ordering, Walsh ordering and cake-cutting ordering. At a sampling rate of $5\%$, the PSNR and SSIM based on the max-projection ordering were 1.1 dB and 0.025 higher, respectively, than those based on the cake-cutting ordering with the best performance in the reference group. Meanwhile, the PSNR and SSIM of our proposed max-projection ordering were merely 2.1 dB and 0.031 lower than those of the ideal ordering, which required the object’s structures to be known in advance. The higher-quality reconstruction at ultralow sampling rates implies a lower radiation dose and less time consumption for data acquisition and computation, which brings great convenience for practical applications.

In noisy simulations, the noise level was set to one order of magnitude higher than the normal background noise. The replacement reconstruction showcased the remarkable accuracy of the reconstruction. At ultralow sampling rates, the retrieved images under different noise levels were almost identical to those in the absence of noise. This demonstrated the noise robustness of the max-projection ordering, especially at low sampling rates.

The orthogonality of the Hadamard matrix results in its Moore–Penrose inverse being equal to its transpose, so the reconstruction time for the image with $64\times 64$ pixels was drastically decreased to milliseconds via simple matrix multiplication, which is of great significance for real-time ghost imaging. In addition, an excellent ordering of the Hadamard matrix could be obtained through only 20 training images, which is beneficial for realistic scenes where the availability of extensive supporting data is limited.

Moving forward, our group will experimentally validate the idea of the max-projection ordering via fabricating a batch of Hadamard masks with gold foils. Moreover, the binarity of Hadamard masks prevents the max-projection ordering from taking full advantage of its strength. Therefore, the max-projection ordering will be attempted to optimize the ordering of nonbinary but orthogonal masks. Numerical simulations will be carried out to provide instructions for the experiments, including the verification of the suitability of the max-projection ordering Hadamard bases in compressed sensing.

In addition to the original advantages of Hadamard masks in terms of easy manufacturing, programmability and rapid refresh rate, the max-projection ordering Hadamard masks provide enhanced reconstruction quality, superior resilience to noise and a remarkable reconstruction time of milliseconds at low sampling rates, laying a solid foundation for the development of real-time ghost imaging.

## Data Availability

The datasets generated during and/or analyzed during the current study are available from the corresponding author on reasonable request. Moreover, the training set of face images in the submitted paper was obtained from an online open-access publication at the following URL: http://mmlab.ie.cuhk.edu.hk/projects/CelebA.html.
